# PK/PD study of vancomycin in enterococcal infections: efficacy and safety assessment under the guidance of TDM

**DOI:** 10.1128/spectrum.00937-26

**Published:** 2026-06-15

**Authors:** Hongyan Zhu, Jingli Liao, Wei Wu, Fenfen Gu, Tong Zhu, Xiaohui Huang, Jian Zhang, Lixia Li

**Affiliations:** 1 Department of Clinical Pharmacy, Xinhua Hospital, School of Medicine, Shanghai Jiao Tong University 12474, Shanghai, China; 2 Dalian Medical University 36674, Dalian, China; 3 Key Laboratory of Cell Differentiation and Apoptosis of National Ministry of Education, Department of Pathophysiology, Shanghai Jiao Tong University School of Medicine 56694, Shanghai, China; Newcastle University, Newcastle Upon Tyne, United Kingdom

**Keywords:** vancomycin, *Enterococcus*, therapeutic drug monitoring (TDM), pharmacokinetics/pharmacodynamics (PK/PD), individualized therapy

## Abstract

Vancomycin is widely utilized in the treatment of *Enterococcus* infections; however, the optimal pharmacokinetic/pharmacodynamic (PK/PD) targets, specifically the 24 h area under the concentration-time curve at steady state (AUC_24h_), and trough concentration (*C*_min_), remain undefined. This study aims to examine the relationship between vancomycin exposure and clinical outcomes in *Enterococcus* infections while establishing PK/PD target values. A retrospective observational analysis was performed on 154 hospitalized patients diagnosed with *Enterococcus* infections treated with vancomycin. Clinical efficacy, acute kidney injury (AKI), 30-day all-cause mortality, and 14-day bacterial clearance were evaluated. Multivariate logistic regression, Cox regression, and receiver operating characteristic curve analysis were employed to identify independent predictors and determine optimal exposure thresholds. The clinical efficacy rate was 72.1%, with an AKI incidence of 20.8%, a 30-day all-cause mortality rate of 14.3%, and a 14-day bacterial clearance rate of 77.9%. Multivariate analysis identified C-reactive protein >100 mg/L, the presence of bloodstream infections, and an AUC_24h_ > 380.00 mg·h/L as independent predictors of clinical efficacy. AUC_24h_ > 540.00 mg·h/L and the Sequential Organ Failure Assessment (SOFA) score were independently associated with the occurrence of AKI. Independent risk factors for 30-day mortality included the SOFA score, surgery, age >60 years, and AUC_24h_ > 580.00 mg·h/L. Intra-abdominal infections and *C*_min_ > 11.00 mg/L were identified as independent predictors of 14-day bacterial clearance. For *Enterococcus spp*., the recommended therapeutic target ranges are AUC_24h_ 370–540 mg·h/L and *C*_min_ 10–14 mg/L. For *Enterococcus faecium*, the optimal therapeutic window is AUC_24h_ 425–560 mg·h/L and *C*_min_ 10–18 mg/L. Optimized vancomycin exposure targets vary by *Enterococcus* species. A stratified PK/PD-guided approach—utilizing a broader initial target when the species is unknown and a refined target range for confirmed *E. faecium*—may enhance therapeutic efficacy while minimizing nephrotoxicity. These findings provide evidence supporting individualized vancomycin dosing in the treatment of enterococcal infections.

**IMPORTANCE:**

*Enterococcus* infections pose a significant treatment challenge, as optimal vancomycin dosing remains poorly defined. This study establishes evidence-based, species-specific exposure targets. For *Enterococcus spp*., the recommended targets are AUC_24h_ 370–540 mg·h/L and *C*_min_ 10–14 mg/L; for *Enterococcus faecium*, a refined window of AUC_24h_ 425–560 mg·h/L and *C*_min_ 10–18 mg/L is proposed. These findings have important implications for individualized dosing and patient outcomes in *Enterococcus* infections.

## INTRODUCTION

The genus *Enterococcus* comprises gram-positive cocci that have become a formidable threat to global healthcare systems (1, 2). Opportunistic pathogens can, under specific conditions, cause severe infections such as infective endocarditis and bacteremia, potentially leading to fatal outcomes (3, 4). Additionally, Enterococci are notorious for their association with hospital-acquired infections and persistently high mortality rates (5–8). The glycopeptide antibiotic vancomycin is commonly used to treat Enterococci infections. Although vancomycin-resistant strains have emerged (9, 10), vancomycin-sensitive strains still constitute the majority in clinical practice (6). Therefore, vancomycin remains the first-line treatment option for *Enterococcus* infections (9). However, the optimal pharmacokinetic/pharmacodynamic (PK/PD) parameters for vancomycin in treating *Enterococcus* infections remain unclear. In clinical practice, treatment standards for methicillin-resistant *Staphylococcus aureus* (MRSA) are often referenced (9, 11). This approach may have limitations, as biological differences between pathogens could make direct application of these standards suboptimal for achieving the best therapeutic outcomes.

Currently, therapeutic drug monitoring (TDM)-based individualized dosing strategies have been proven to significantly improve vancomycin efficacy and reduce adverse effects in MRSA infections (12–14). Nevertheless, *Enterococcus* and *Staphylococci* exhibit substantial differences in biological characteristics and pathogenic mechanisms, making direct adoption of MRSA-derived PK/PD targets potentially ineffective for optimal *Enterococcus* treatment (3, 15, 16). Multiple studies have observed distinct differences in clinical responses and drug tolerance between *Enterococcus* and MRSA infection patients treated with vancomycin (17, 18). These differences may stem from *Enterococcus* unique cell wall structure, biofilm-forming capability, and site-specific pharmacokinetic properties. Compared to *Staphylococcus aureus*, the peptidoglycan layer of *Enterococcus* contains more low-branched cross-bridge peptides, which not only reduce the affinity of β-lactam antibiotics but may also affect the penetration efficiency of large glycopeptide molecules such as vancomycin. Additionally, its intrinsic tolerance and acquired resistance capabilities render traditional PK/PD targets, which are based on staphylococcal data, potentially inapplicable to *Enterococcus* (19). The first comprehensive vancomycin treatment guidelines published in 2009 (20) and the updated 2020 consensus guidelines for vancomycin concentration monitoring in MRSA infections recommend trough concentrations (*C*_min_) of 10–20 mg/L and AUC/MIC ≥ 400. These standards are primarily based on MRSA infection data (11, 21). However, the applicability of these parameters to *Enterococcus* infections lacks sufficient evidence-based medical support. Notably, *Enterococcus* infections frequently occur in specific sites such as the urinary tract and intra-abdominal sites, where drug penetration may significantly impact treatment efficacy (2, 19). Furthermore, the nephrotoxicity risk profile of vancomycin in *Enterococcus*-infected patients requires further evaluation.

With the advancement of precision medicine, PK/PD-based individualized dosing strategies have attracted considerable attention (22–24). However, reliable PK/PD targets for vancomycin in *Enterococcus* infections have not been established, and the scarcity of related research limits rational drug use. This study aimed to explore the relationship between vancomycin concentrations and clinical efficacy/adverse reactions through retrospective analysis of clinical data from *Enterococcus*-infected patients, establishing PK/PD target ranges specifically for *Enterococcus* infections. The findings will provide scientific evidence for developing individualized dosing regimens and have significant implications for improving the efficacy and safety of vancomycin in treating *Enterococcus* infections. Additionally, this study will contribute new evidence-based medical data to refine vancomycin TDM guidelines.

## MATERIALS AND METHODS

### Study design and participants

**Fig 1:**
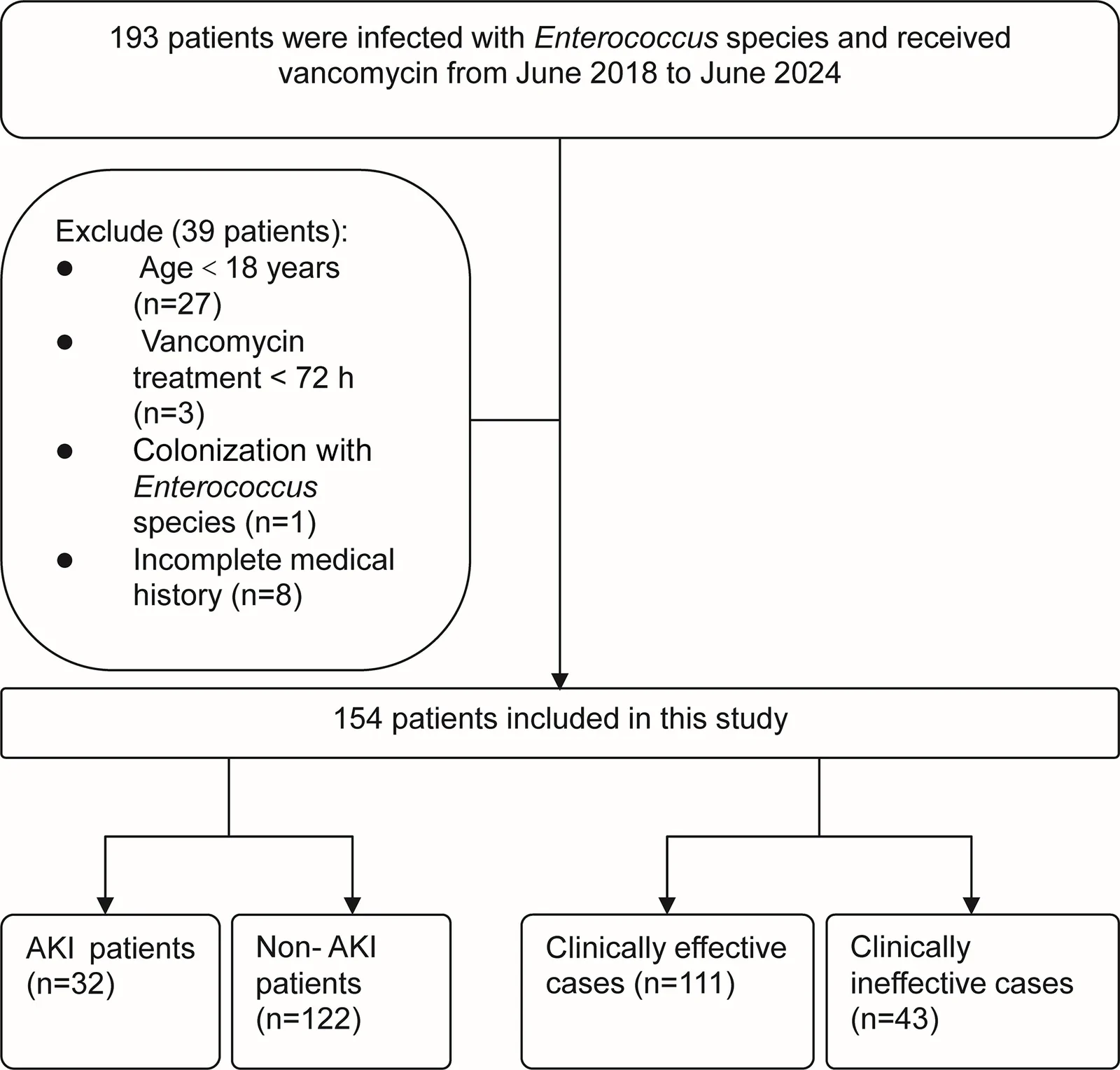
Flowchart of patient selection. AKI, acute kidney injury.

This retrospective observational study enrolled 154 patients with *Enterococcus* infections hospitalized at Xinhua Hospital Affiliated to Shanghai Jiao Tong University School of Medicine between January 2021 and September 2024 (Fig. 1).

#### Inclusion criteria

Age ≥18 years, microbiologically confirmed *Enterococcus* infections, intravenous vancomycin treatment, and at least one vancomycin peak-trough concentration monitoring. Exclusion criteria: vancomycin treatment duration <72 h or incomplete clinical data.

### Data collection

The following data were collected through the hospital electronic medical record system: (i) Demographic characteristics: age, sex, and weight. (ii) Clinical features: infection site, Sequential Organ Failure Assessment (SOFA) score, comorbidities, surgical history, mechanical ventilation status, and *Enterococcus* species. (iii) Laboratory parameters: liver/kidney function, C-reactive protein (CRP), procalcitonin (PCT), and blood routine index. (iv) Treatment-related data: vancomycin dosing regimen and combination therapies. (v) Efficacy and safety outcomes: clinical response, acute kidney injury (AKI) incidence, 30-day mortality, and bacterial clearance rate.

### Vancomycin concentration monitoring and AUC estimation

All patients received intravenous vancomycin infusion, with dosing adjusted based on renal function and monitored concentrations. Steady-state trough (*C*_min_) and peak (*C*_max_) samples were collected before the third to fifth dose (30 min pre-dose and 1 h post-infusion, respectively). Vancomycin concentrations were measured by high-performance liquid chromatography as part of routine TDM practice. Each measurement included two samples for *C*_min_ and *C*_max_ determination. The AUC_24h_ was calculated using the two-point method (peak and trough concentrations) formula in this study (25)

Based on the calculated AUC_24h_, trough concentration, renal function, and the patient’s clinical status, clinical pharmacists provided individualized dose adjustment recommendations, including modifications to both dose and dosing intervals. These recommendations were documented in pharmaceutical records and communicated to the attending physicians through the electronic medical record system or written consultation reports. Attending physicians made the final prescribing decisions by integrating the clinical pharmacists’ recommendations with the patient’s overall clinical condition. Consequently, the vancomycin concentrations and dosing regimens documented in this study reflect real-world clinical outcomes under TDM guidance.

As a retrospective observational study, this investigation did not predefine an interventional protocol, nor did it require physician adherence to the pharmacists’ recommendations. The study recorded vancomycin concentrations obtained from routine clinical monitoring alongside corresponding clinical outcomes, aiming to evaluate the relationship between vancomycin exposure and clinical outcomes within the framework of real-world TDM practice.

### Study endpoints

The primary endpoints included the analysis of factors influencing the clinical efficacy of vancomycin and the occurrence of AKI, as well as determination of their thresholds. The secondary endpoints included the 30-day all-cause mortality rate of patients, the bacterial clearance rate, etc.

#### Definitions

##### Clinical efficacy

Clinical efficacy was assessed 72 h after the initiation of vancomycin treatment. Patients were classified as clinically effective if they met all of the following criteria: (i) Temperature normalization: Body temperature ≤37.5°C sustained for at least 24 h. (ii) Improvement in infection biomarkers: CRP level decreased by ≥30% from baseline or decreased to ≤50 mg/L; and/or PCT level decreased by ≥50% from baseline or decreased to ≤0.5 ng/mL. (iii) Normalization or improvement in white blood cell count: White blood cell count within the range of 4.0–12.0 × 10⁹/L, or a decrease of ≥30% from baseline. (iv) Stable or improved organ function: SOFA score did not increase from baseline or decrease. Patients who did not meet all of the above criteria were classified as clinically ineffective.

##### Acute kidney injury

For adults, the creatinine clearance rate is calculated using the Cockcroft-Gault formula. Acute kidney injury is diagnosed when there is a continuous increase of 0.3 mg/dL (26.5 μmol/L) in serum creatinine from the baseline value, along with a 50% increase during the course of treatment.

##### 30-day all-cause mortality

This is recorded starting from the initiation of vancomycin treatment. It refers to the occurrence of death due to any cause within 30 days of the treatment start.

##### 14-day bacterial clearance rate

Recorded from the first detection of the pathogen, bacterial clearance is achieved when the bacterial culture turns negative or when the bacterial culture shows the growth of other bacteria, while the original pathogen no longer appears. Non-clearance is defined as the continued presence of the original pathogen in the bacterial culture.

### Statistical analysis

SPSS version 27.0 (IBM Corp, Armonk, NY, USA) was used for statistical analysis. Continuous variables were expressed as median (interquartile range, IQR), and categorical variables were expressed as counts (percentages). Multivariate logistic regression identified independent factors for clinical efficacy, bacterial clearance, and AKI (expressed as odds ratios, ORs). Cox regression analyzed 30-day mortality risk factors (hazard ratios, HRs). Receiver operating characteristic (ROC) curve analysis with Youden index determined optimal *C*_min_ and AUC_24h_ thresholds. Kaplan-Meier analysis assessed 30-day mortality and vancomycin-associated AKI. GraphPad Prism 8 was used for graphical presentation. *P* < 0.05 was considered statistically significant.

## RESULTS

### Epidemiological characteristics

Among 193 initially screened patients, 154 met inclusion criteria (Fig. 1). Median age was 70 years (IQR: 60–79), with 61.7% male and 69.5% ICU admitted. Primary infection sites were intra-abdominal (39.0%, 60/154), bloodstream (37%, 57/154), and urinary tract (28.6%, 44/154). Common comorbidities included hypertension (42.9%, 66/154), malignancy (39.6%, 61/154), and heart disease (29.2%, 45/154). Combination antibiotics were used in 89.6% (138/154), with carbapenems accounting for 50%. *Enterococcus* species included *Enterococcus faecium* (63%, 97/154), *Enterococcus faecalis* (41.6%, 64/154), *Enterococcus avium* (4.5%, 7/154), and *Enterococcus gallinarum* (2.6%, 4/154). From 154 patients, a total of 161 isolates of *Enterococcus faecium* and *Enterococcus faecalis* were recovered, including 147 cases with a single strain and 7 cases with concurrent infection involving two or more strains. The minimum inhibitory concentration (MIC) range of vancomycin for all strains was found to be 0.5–2 mg/L, with MIC_50_ and MIC_90_ values recorded at 0.75 mg/L and 1.5 mg/L, respectively. The overall distribution did not reveal any exceptionally high values that would indicate resistance or intermediate susceptibility. This trend aligns with the status of “maintenance of high susceptibility,” as evidenced by the susceptibility rate of *enterococci* to vancomycin, which exceeds 95%, as reported by the CHINET surveillance network in 2023 (26). This indicates that the strains examined in this study are representative of the broader population. Among 154 patients, 83 underwent two or more pharmacokinetic assessments, yielding 528 blood samples analyzed in 264 separate tests. *C*_min_: 11.90 mg/L (IQR: 8.20–17.30), *C*_max_: 23.50 mg/L (IQR: 17.90–31.20), and AUC_24h_: 431.90 mg·h/L (IQR: 317.30–570.70). The demographic and clinical characteristics of the patients are detailed in Table 1.

**TABLE 1:** Patient characteristics^*a*^^,^^*b*^

Parameters	Variable
Age, years, median (IQR)	70.0 (60.0–79.0)
Male, *n* (%)	95.0 (61.7)
Female, *n* (%)	59.0 (38.3)
Body weight, kg, median (IQR)	60.0 (50.0–65.0)
ICU admission, *n* (%)	107.0 (69.5)
Mechanical ventilation, *n* (%)	96.0 (62.3)
CRRT, *n* (%)	19.0 (12.3)
SOFA, median (IQR)	3.0 (1.0–5.0)
Duration of hospitalization, days, median (IQR)	38.5 (23.0–61.8)
Comorbidities, *n* (%)	
Heart disease	45.0 (29.2)
Diabetes	38.0 (24.7)
Hypertension	66.0 (42.9)
Kidney diseases	18.0 (11.7)
Liver diseases	21.0 (13.6)
Malignancy	61.0 (39.6)
Other^*c*^	27.0 (17.5)
Enterococcal species, *n* (%)	
*E. faecalis*	64.0 (41.6)
*E. faecium*	97.0 (63.0)
*E. avium*	7.0 (4.5)
*E. gallinarum*	4.0 (2.6)
MIC, median (IQR)	0.8 (0.5–1.0)
*E. faecalis*	1.0 (1.0–1.0)
*E. faecium*	0.5 (0.5–1.0)
*E. avium*	0.5 (0.5–0.5)
*E. gallinarum*	0.8 (0.5–1.8)
*E. faecalis* MIC, *n* (%)	
≤0.5, *n* (%)	11.0 (17.2)
1, *n* (%)	38.0 (59.4)
≥2, *n* (%)	15.0 (23.4)
*E. faecium* MIC, *n* (%)	
≤0.5, *n* (%)	66.0 (68.0)
1, *n* (%)	31.0 (32.0)
Source of enterococcal infection, *n* (%)	
Bloodstream infection	57.0 (37.0)
Pulmonary infection	11.0 (7.1)
Wound infection	12.0 (7.8)
Urinary tract infection	44.0 (28.6)
Intra-abdominal infection	60.0 (39.0)
Vancomycin treatment, median (IQR)	
Duration days, days	12.0 (9.0–17.0)
Daily dose, mg	16,500.0 (9,000.0–28,375.0)
Daily dose/weight, mg/kg/day	25.0 (17.1–30.0)
Concomitant antibiotics, *n* (%)	
Imipenem cilastatin	23.0 (14.9)
Meropenem	54.0 (35.1)
Piperacillin tazobactam	26.0 (16.9)
Levofloxacin	14.0 (9.1)
Amikacin	4.0 (2.6)
Fosfomycin	5.0 (3.2)
Polymyxin	8.0 (5.2)
Other^*d*^	67.0 (43.5)
Laboratory data	
C-reactive protein, mg/L	99.5 (43.3–160.0)
White blood cell, 10^9^/L	12.4 (8.3–17.7)
Platelets, 10^9^/L	170.5 (92.5–236.5)
Hemoglobin, g/L	93.0 (80.8–110.0)
Red blood cell, 10^9^/L	3.2 (2.7–3.6)
Procalcitonin, ng/mL	1.4 (0.2–6.5)
Albumin, g/L	31.8 (27.5–36.1)
Alanine transaminase, U/L	25.0 (15.0–51.0)
Alkaline phosphatase, U/L	92.5 (66.8–138.0)
Total bilirubin, μmol/L	19.3 (12.8–33.6)
Direct bilirubin, μmol/L	3.0 (0.0–12.2)
Blood urea nitrogen, mmol/L	8.0 (5.0–13.0)
Serum creatinine, μmol/L	64.9 (50.8–124.8)
AUC_24h_, mg·h/L	431.9 (317.3–570.7)
*C*_min_, mg/L	11.9 (8.2–17.3)
*C*_max_, mg/L	23.5 (17.9–31.2)

^*a*^ IQR, interquartile range; ICU, intensive care unit; CRRT, continuous renal replacement therapy; SOFA, sequential organ failure assessment; AUC_24h_, 24 h area under the concentration-time curve at steady state; *C*_min_, trough concentration; *C*_max_, peak concentration; MIC, minimal inhibitory concentration.

^*b*^ Bacterial names are shown in italics.

^*c*^ Systemic lupus erythematosus, chronic bronchitis, rheumatoid arthritis, and clinical depression.

^*d*^ Cefepime, cefmetazole, cefoperazone sulbactam, linezolid, voriconazole, and fluconazole.

### Analysis of factors affecting the clinical efficacy of vancomycin

**Fig 2:**
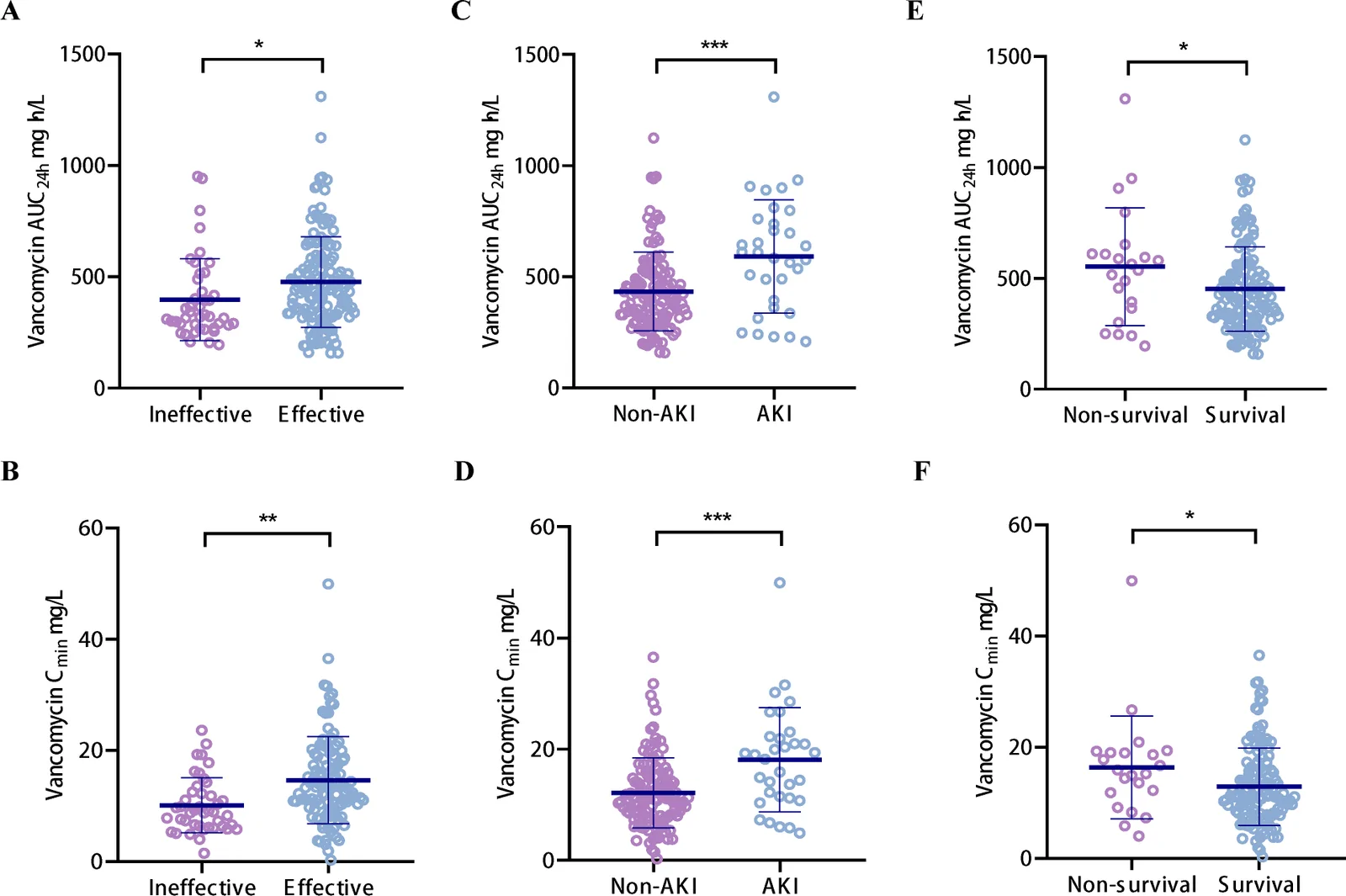
Scatterplot of vancomycin AUC_24h_ and *C*_min_ classified according to outcome. (**A**) AUC_24h_ and (**B**) *C*_min_ for clinical efficacy. (**C**) AUC_24h_ and (**D**) *C*_min_ for AKI. (**E**) AUC_24h_ and (**F**) *C*_min_ for survival. *C*_min_, trough concentration; AUC_24h_, 24 h area under the concentration-time curve at steady state; AKI, acute kidney injury. ^*^P < 0.05, ^**^P < 0.01, and ^***^P < 0.001.

The efficacy rate was 72.1% (111/154). The effective group showed significantly higher median AUC_24h_ (458.5 vs 321.7 mg·h/L, *P* = 0.011; Fig. 2A) and *C*_min_ (13.40 vs 8.94 mg/L, *P* = 0.001; Fig. 2B) than the ineffective group. Univariate analysis identified CRP >100 mg/L (OR: 0.373; 95% CI: 0.168–0.826; *P* = 0.015), bloodstream infections (OR: 0.440; 95% CI: 0.214–0.903; *P* = 0.025), *C*_min_ (OR: 1.123; 95% CI: 1.049–1.203; *P* = 0.001), and AUC_24h_ (OR: 1.003; 95% CI: 1.001–1.005; *P* = 0.011) as significant factors. Multivariate analysis confirmed CRP >100 mg/L (OR: 0.359; 95% CI: 0.155–0.830; *P* = 0.017), bloodstream infections (OR: 0.434; 95% CI: 0.190–0.999; *P* = 0.047), and AUC_24h_ >380.00 mg·h/L (OR: 3.320; 95% CI: 1.464–7.528; *P* = 0.004) as independent predictors (Table 2).

**TABLE 2:** Univariate and multivariate logistic regression models for clinical efficacy of vancomycin^*a*^^,^^*b*^

Parameters	Effective *n* = 111	Failure *n* = 43	OR (95% CI)	*P* value
Univariate analysis				
Age, years, median (IQR)	70 (56, 82)	69 (61, 75)	1.006 (0.985–1.027)	0.590
Comorbidities, *n* (%)				
Heart disease	33 (29.7)	12 (27.9)	1.093 (0.501–2.386)	0.823
Diabetes	28 (25.2)	10 (23.3)	1.055 (0.698–1.595)	0.799
Hypertension	48 (43.2)	18 (41.9)	1.019 (0.804–1.292)	0.876
Kidney diseases	15 (13.5)	3 (7.0)	1.201 (0.870–1.660)	0.266
Liver diseases	16 (14.4)	5 (11.6)	1.051 (0.848–1.302)	0.652
Malignancy	41 (36.9)	20 (46.5)	0.936 (0.831–1.054)	0.277
Test index before vancomycin treatment				
CRP, mg/L, median (IQR)	77 (41,160)	159 (62,160)	0.994 (0.988–1.000)	0.058
CRP > 100 vs ≤100, *n* (%)	42 (44.7)	26 (68.4)	0.373 (0.168–0.826)	**0.015**
White blood cell, 10^9^/L, median (IQR)	11.2 (8.0, 15.9)	16.7 (12.5, 22.1)	0.969 (0.932–1.008)	0.114
Procalcitonin, ng/mL, median (IQR)	1.2 (0.2, 6.2)	2.9 (0.2, 6.6)	1.009 (0.985–1.035)	0.456
eGFR, mL/min, median (IQR)	69.6 (35.0, 106.6)	69.0 (46.4, 97.1)	0.999 (0.992–1.007)	0.859
SOFA, median (IQR)	3 (1, 5)	4 (1, 6)	0.958 (0.845–1.087)	0.508
Mechanical ventilation, *n* (%)	64 (57.7)	32 (74.4)	0.468 (0.214–1.023)	0.057
CRRT, *n* (%)	13 (11.7)	6 (14.0)	0.818 (0.29–2.311)	0.705
Surgery, *n* (%)	89 (80.2)	32 (74.4)	1.391 (0.607–3.186)	0.436
Infection type, *n* (%)				
Bloodstream infection	35 (31.5)	22 (51.2)	0.440 (0.214–0.903)	**0.025**
Pulmonary infection	7 (6.3)	4 (9.3)	0.810 (0.427–1.538)	0.520
Wound infection	10 (9.0)	2 (4.7)	1.266 (0.752–2.130)	0.374
Urinary tract infection	32 (28.8)	12 (27.9)	1.009 (0.863–1.180)	0.910
Intra-abdominal infection	44 (39.6)	16 (37.2)	1.017 (0.901–1.148)	0.781
Vancomycin therapeutic regimen				
Single dose of vancomycin, mg/kg, median (IQR)	8.3 (7.5, 10.0)	8.3 (8.2, 10.3)	0.934 (0.829–1.053)	0.264
Daily dose, mg/kg, median (IQR)	24.2 (16.7, 30.0)	25.0 (19.2, 32.0)	0.988 (0.952–1.025)	0.516
Concomitant antibiotics, *n* (%)				
Imipenem cilastatin	16 (14.4)	7 (16.3)	0.866 (0.329–2.279)	0.771
Meropenem	36 (32.4)	18 (41.9)	0.667 (0.323–1.376)	0.273
Piperacillin tazobactam	15 (13.5)	11 (25.6)	0.455 (0.19–1.09)	0.077
Levofloxacin	9 (8.1)	5 (11.6)	0.671 (0.211–2.128)	0.498
Amikacin	2 (1.8)	2 (4.7)	0.376 (0.051–2.759)	0.336
Fosfomycin	2 (1.8)	3 (7.1)	0.239 (0.038–1.481)	0.124
TDM, median (IQR)				
*C*_min_, mg/L	13.4 (10.0, 19.0)	8.9 (6.4, 12.5)	1.123 (1.049–1.203)	**0.001**
*C*_min_ > 9.5 vs ≤9.5	86 (77.5)	19 (44.2)	4.345 (2.055–9.187)	**<0.001**
AUC_24h_, mg·h/L	458.5 (353.4, 594.9)	321.7 (282.8, 489.0)	1.003 (1.001–1.005)	**0.011**
AUC_24h_ > 380 vs ≤380	75 (67.6)	16 (37.2)	3.516 (1.686–7.332)	**0.001**
AUC_24h_/MIC	633.3 (380.4, 960.4)	527.0 (314.3, 730.1)	1.001 (1.000–1.002)	0.064
*C*_max_, mg/L	24.7 (18.0, 32.0)	20.0 (16.6, 26.9)	1.028 (0.990–1.068)	0.148
MIC, median (IQR)	1.0 (0.5, 1.0)	0.5 (0.5, 1.0)	1.066 (0.524–2.169)	0.860
Pathogenic bacteria species, *n* (%)				
*E. faecalis*	42 (37.8)	22 (51.2)	0.581 (0.286–1.182)	0.134
MIC ≥ 1 vs ≤0.5	35 (83.3)	18 (81.8)	1.111 (0.287–4.302)	0.879
*E. faecium*	72 (64.9)	25 (58.1)	1.153 (0.804–1.653)	0.439
MIC *≥* 1 vs ≤0.5	25 (34.7)	6 (24.0)	1.684 (0.596–4.757)	0.325
*E. avium*	4 (3.6)	3 (7)	0.793 (0.474–1.325)	0.376
*E. gallinarum*	4 (3.6)	0 (0)	159.6 (0–0)	0.999
Multivariate analysis				
CRP > 100 vs <100, *n* (%)			0.359 (0.155–0.830)	**0.017**
Bloodstream infection			0.434 (0.190–0.999)	**0.047**
AUC_24h_ > 380 vs ≤380			3.320 (1.464-7.528)	**0.004**

^*a*^ *P *values < 0.05 are shown in bold; bacterial names are shown in italics. For all binary comparisons presented as “≥/> X vs ≤/< Y” in the tables, only the number and percentage of isolates in the higher category (≥/> X) are shown. The remaining isolates fall into the complementary lower category (≤/< Y), with percentages calculated based on the total number of strains in each respective group.

^*b*^ IQR, interquartile range; C-reactive protein, CRP; eGFR, estimated glomerular filtration rate; SOFA, sequential organ failure assessment; CRRT, continuous renal replacement therapy; TDM, therapeutic drug concentration monitoring; *C*_min_, trough concentration; AUC_24h_, 24 h area under the concentration-time curve at steady state; *C*_max_, peak concentration; MIC, minimal inhibitory concentration; peak concentration.

### Analysis of factors affecting vancomycin-induced acute kidney injury

AKI occurred in 20.8% (32/154). AKI patients had significantly higher median AUC_24h_ (596.40 vs 404.90 mg·h/L, *P* < 0.001; Fig. 2C) and *C*_min_ (18.6 vs 11.1 mg/L, *P* < 0.001; Fig. 2D). Univariate analysis associated SOFA score, mechanical ventilation, *C*_min_, *C*_max_, AUC_24h_, and AUC_24h_/MIC with AKI. Multivariate analysis identified SOFA score (OR: 1.224; 95% CI: 1.055–1.420; *P* = 0.008) and AUC_24h_ > 540.00 mg·h/L (OR: 5.511; 95% CI: 2.322–13.079; *P* < 0.001) as independent risk factors (Table 3).

**TABLE 3:** Analysis of factors influencing acute kidney injury during vancomycin treatment^*a*^^,^^*b*^

Parameters	AKI *n* = 32	Non-AKI *n* = 122	OR (95% CI)	*P* value
Univariate analysis				
Age, years, median (IQR)	70.0 (60.0, 78.3)	71.5 (50.0, 82.0)	0.997 (0.974–1.021)	0.805
Test index before vancomycin treatment				
White blood cell, 10^9^/L, median (IQR)	13.0 (11.0, 20.5)	12.5 (8.4, 17.4)	1.035 (0.993–1.079)	0.103
CRP, mg/L, median (IQR)	111 (42.5, 160.0)	107 (49.0, 160.0)	1.0004 (0.9935–1.0073)	0.917
Creatinine, mmol/L, median (IQR)	60.0 (50.6, 123.4)	69.1 (49.5, 123.3)	1.0001 (0.9971–1.0031)	0.955
SOFA, median (IQR)	3 (1, 5)	4.5 (3, 7)	1.255 (1.089–1.447)	**0.002**
Mechanical ventilation, *n* (%)	25 (78.1)	71 (58.2)	2.565 (1.031–6.386)	**0.043**
CRRT, *n* (%)	3 (9.4)	16 (13.1)	0.685 (0.187–2.514)	0.569
Vancomycin therapeutic regimen, median (IQR)				
Single dose of vancomycin, mg/kg	8.3 (7.5, 10.0)	8.4 (7.8, 10.0)	0.886 (0.748–1.050)	0.163
Daily dose, mg/kg	22.5 (15.1, 28.9)	25.0 (18.5, 30.3)	0.967 (0.927–1.010)	0.122
Concomitant antibiotics, *n* (%)				
Imipenem cilastatin	3 (9.4)	20 (16.4)	0.528 (0.146–1.901)	0.328
Meropenem	14 (43.8)	40 (32.8)	1.594 (0.721–3.528)	0.250
Piperacillin tazobactam	8 (25.0)	18 (14.8)	1.926 (0.750–4.947)	0.173
Levofloxacin	2 (6.3)	12 (9.8)	0.611 (0.130–2.880)	0.534
Polymyxin	4 (12.5)	4 (3.3)	4.214 (0.993–17.892)	0.051
MIC, median (IQR)	0.5 (0.5, 1.0)	1.0 (0.5, 1.0)	1.153 (0.552–2.408)	0.704
Pathogenic bacteria species, *n* (%)				
*E. faecalis*	10 (31.3)	54 (44.3）	1.747 (0.763–4.000）	0.187
MIC *≥* 1 vs ≤0.5	8 (80.0)	45 (83.3）	0.800 (0.145–4.409）	0.798
*E. faecium*	22 (68.8)	75 (61.5）	1.174 (0.775–1.780）	0.449
MIC *≥ 1* vs ≤0.5	7 (31.8）	24 (32.0）	0.992 (0.358–2.75）	0.987
*E. avium*	1 (3.1）	6 (4.9）	0.854 (0.417–1.752）	0.667
*E. gallinarum*	2 (6.3）	2 (1.6）	1.414 (0.858–2.332）	0.174
TDM, median (IQR)				
*C*_min_, mg/L	18.6 (11.3, 22.2)	11.1 (7.9, 15.5)	1.109 (1.048–1.173)	**<0.001**
*C*_min_ > 15 vs ≤15, *n* (%)	18 (56.3)	33 (27.1)	3.468 (1.551–7.753)	**0.002**
AUC_24h_, mg·h/L	596.4 (369.6, 754.3)	404.9 (315.0, 513.6)	1.004 (1.002–1.005)	**<0.001**
AUC_24h_ > 540 vs ≤540, *n* (%)	19 (59.4)	24 (19.7)	5.968 (2.590–13.753)	**<0.001**
*C*_max_, mg/L	31.9 (20.2, 35.8)	21.8 (17.9, 28.4)	1.052 (1.015–1.089)	**0.005**
*C*_max_ > 35 vs ≤35, *n* (%)	10 (31.3)	12 (9.8)	4.167 (1.602–10.838)	**0.003**
AUC_24h_/MIC	850.2 (426.3, 1,269.9)	536.7 (353.4, 814.6)	1.0014 (1.0004–1.0023)	**0.004**
AUC_24h_/MIC > 595 vs ≤595, *n* (%)	20 (62.5)	52 (42.62)	2.244 (1.008–4.996)	**0.048**
Multivariate analysis				
SOFA, median (IQR)			1.224 (1.055–1.420)	**0.008**
AUC_24h_ > 540 vs ≤540, *n* (%)			5.511 (2.322–13.079)	**<0.001**

^*a*^ *P *values < 0.05 are shown in bold; bacterial names are shown in italics. For all binary comparisons presented as "≥/> X vs ≤/< Y" in the tables, only the number and percentage of isolates in the higher category (≥/> X) are shown. The remaining isolates fall into the complementary lower category (≤/< Y), with percentages calculated based on the total number of strains in each respective group.

^*b*^ AKI, acute kidney injury; IQR, interquartile range; C-reactive protein, CRP; eGFR, estimated glomerular filtration rate; SOFA, sequential organ failure assessment; CRRT, continuous renal replacement therapy; TDM, therapeutic drug concentration monitoring; *C*_min_, trough concentration; AUC_24h_, 24 h area under the concentration-time curve at steady state; *C*_max_, peak concentration; MIC, minimal inhibitory concentration; peak concentration.

### Analysis of the factors affecting the 30-day all-cause mortality of vancomycin

Thirty-day all-cause mortality was 14.3% (22/154). Univariate Cox regression identified age, SOFA score, mechanical ventilation, surgery, *C*_max_, *C*_min_, and AUC_24h_ as significant factors. Multivariate analysis established SOFA score (HR: 1.169; 95% CI: 1.037–1.317; *P* = 0.011), surgery (HR: 0.336; 95% CI: 0.141–0.802; *P* = 0.014), age >60 years (HR: 10.669; 95% CI: 1.422–80.075; *P* = 0.021), and AUC_24h_ > 580.00 mg·h/L (HR: 2.495; 95% CI: 1.064–5.85; *P* = 0.035) as independent predictors (Table 4). Kaplan-Meier survival curves are shown in Fig. S1.

**TABLE 4:** Univariate and multivariate analysis of 30-day all-cause mortality^*a*^^,^^*b*^

Parameters	HR	95% CI	*P* value
Univariate analysis			
Age, years, median (IQR)	1.039	1.007–1.073	**0.017**
Age > 60 vs ≤60, *n* (%)	9.054	1.218–67.32	**0.031**
Comorbidities, *n* (%)			
Heart disease	1.717	0.734–4.018	0.213
Diabetes	1.233	0.788–1.932	0.359
Hypertension	1.039	0.786–1.375	0.788
Kidney diseases	1.065	0.786–1.444	0.684
Liver diseases	0.775	0.519–1.158	0.214
Malignancy	1.045	0.908–1.202	0.539
Test index before vancomycin treatment			
White blood cell, 10^9^/L, median (IQR)	1.023	0.981–1.068	0.288
C-reactive protein, mg/L, median (IQR)	1.004	0.996–1.011	0.358
Procalcitonin, ng/mL, median (IQR)	1.009	0.989–1.029	0.376
Albumin, g/L	0.970	0.901–1.045	0.425
Platelets, 10^9^/L,	0.996	0.992–1.001	0.099
eGFR, mL/min, median (IQR)	0.992	0.982–1.003	0.152
SOFA, median (IQR)	1.186	1.040–1.353	**0.011**
Mechanical ventilation, *n* (%)	4.032	1.193–13.628	**0.025**
CRRT, *n* (%)	1.670	0.565–4.935	0.3535
Surgery, *n* (%)	0.353	0.151–0.827	**0.016**
MIC, median (IQR)	1.378	0.714–2.659	0.334
Infection type, *n* (%)			
Bloodstream infection	2.152	0.930–4.982	0.074
Pulmonary infection	1.483	0.806–2.726	0.205
Wound infection	0.818	0.419–1.597	0.557
Urinary tract infection	0.987	0.818–1.191	0.895
Intra-abdominal infection	0.906	0.775–1.059	0.216
Vancomycin therapeutic regimen			
Single dose of vancomycin, mg/kg, median (IQR)	1.028	0.888–1.19	0.711
Daily dose, mg/kg, median (IQR)	0.980	0.937–1.024	0.365
Concomitant antibiotics, *n* (%)			
Imipenem cilastatin	1.232	0.417–3.64	0.706
Meropenem	1.288	0.551–3.014	0.559
Piperacillin tazobactam	0.776	0.23–2.623	0.683
Levofloxacin	1.023	0.239–4.377	0.975
Fosfomycin	3.313	0.771–14.23	0.107
Polymyxin	0.829	0.111–6.161	0.854
TDM, median (IQR)			
*C*_min_, mg/L	1.0530	1.005–1.103	**0.029**
*C*_min_ > 15 vs ≤15, *n* (%)	2.622	1.132–6.069	**0.024**
AUC_24h_, mg·h/L	1.0020	1.0002–1.0037	**0.025**
AUC_24h_ > 580 vs ≤580, *n* (%)	3.000	1.296–6.945	**0.010**
*C*_max_, mg/L	1.0412	1.008–1.076	**0.015**
AUC_24h_/MIC	1.0004	0.9995–1.0014	0.354
Pathogenic bacteria species, *n* (%)			
*E. faecalis*	1.434	0.622–3.309	0.398
*E. faecium*	0.750	0.494–1.139	0.178
*E. avium*	1.332	0.821–2.164	0.246
*E. gallinarum*	1.143	0.692–1.887	0.602
Multivariate analysis			
AUC_24h_ > 580 vs ≤580, *n* (%)	2.495	1.064–5.85	**0.035**
Surgery	0.336	0.141–0.802	**0.014**
SOFA	1.169	1.037–1.317	**0.011**
Age > 60 vs ≤60, *n* (%)	10.669	1.422–80.075	**0.021**

^*a*^ *P *values < 0.05 are shown in bold; bacterial names are shown in italics. For all binary comparisons presented as “≥/> X vs ≤/< Y” in the tables, only the number and percentage of isolates in the higher category (≥/> X) are shown. The remaining isolates fall into the complementary lower category (≤/< Y), with percentages calculated based on the total number of strains in each respective group.

^*b*^ IQR, interquartile range; eGFR, estimated glomerular filtration rate; SOFA, sequential organ failure assessment; CRRT, continuous renal replacement therapy; TDM, therapeutic drug concentration monitoring; *C*_min_, trough concentration; AUC_24h_, 24 h area under the concentration-time curve at steady state; *C*_max_, peak concentration; MIC, minimal inhibitory concentration; peak concentration.

### Analysis of factors affecting 14-day bacterial clearance of vancomycin

The clearance rate was 77.9% (120/154). Univariate analysis associated *C*_min_ (OR: 1.091; 95% CI: 1.019–1.169; *P* = 0.013) and AUC_24h_ (OR: 1.003; 95% CI: 1.000–1.005; *P* = 0.029) with clearance. Multivariate analysis identified intra-abdominal infections (OR: 0.444; 95% CI: 0.199–0.991; *P* = 0.048) and *C*_min_ > 11.00 mg/L (OR: 2.566; 95% CI: 1.151–5.781; *P* = 0.021) as independent factors (Table S1).

### The optimal *C*_min_ and AUC_24h_ targets for the clinical efficacy and safety of vancomycin in the treatment of *Enterococcus* infections

**Fig 3:**
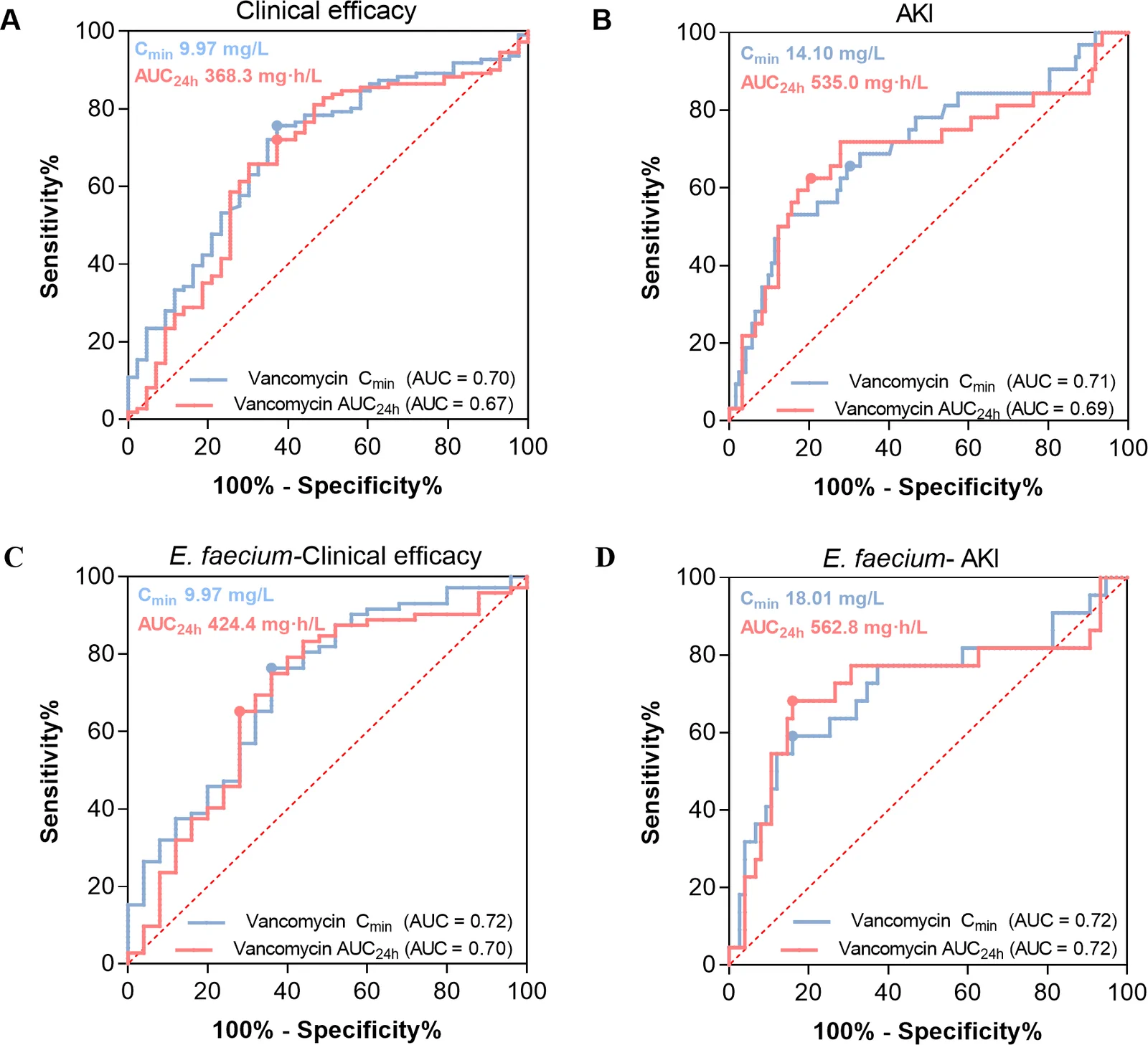
For *Enterococcus* spp, ROC curve of the C_min_ and AUC_24h_ of vancomycin for (**A**) clinical efficacy and (**B**) AKI. For *E. faecium*, ROC curve of the *C*_min_ and AUC_24h_ of vancomycin for (**C**) clinical efficacy and (**D**) AKI. *C*_min_, trough concentration; AUC_24h_, 24 h area under the concentration-time curve at steady state; AKI, acute kidney injury.

The ROC curve analysis showed that, based on the clinical remission rate (Fig. 3A), the critical value *C*_min_ ≥ 9.97 mg/L was determined (sensitivity: 75.7%; specificity: 62.8%), with AUC_24h_ ≥ 368.30 mg·h/L (sensitivity: 71.2%; specificity: 62.8%). Based on AKI (Fig. 3B), the critical value *C*_min_ ≥ 14.10 mg/L was determined (sensitivity: 65.6%; specificity: 70.5%), with AUC_24h_ ≥ 535.00 mg·h/L (sensitivity: 62.5%; specificity: 80.3%). Based on ROC analysis and for clinical convenience, we propose a vancomycin therapeutic target range of AUC_24h_ 370–540 mg·h/L and *C*_min_ 10–14 mg/L for the treatment of these infections. This range optimally balances therapeutic efficacy with the risk of nephrotoxicity. The median *C*_min_ and AUC_24h_ of patients with AKI were significantly higher than those without AKI (Fig. 2C and D). Subgroup analysis showed that in the Kaplan-Meier (log-rank, *P* < 0.001, Fig. S2A) analysis, the AKI incidence in the group with AUC_24h_ > 535.00 mg·h/L (45.5%) was significantly higher than that in the group with AUC_24h_ ranging from 368.30 to 535.00 mg·h/L (8.0%) and the group with AUC_24h_ < 368.30 mg·h/L (13.3%). For the Kaplan-Meier analysis of *C*_min_ (log-rank, *P* < 0.001, Fig. S2B), the AKI incidence in the group with *C*_min_ >14.10 mg/L (36.8%) was significantly higher than that in the group with *C*_min_ ranging from 9.97 to 14.10 mg/L (14.0%) and the group with *C*_min_ < 14.10 mg/L (9.3%).

### Analysis of PK/PD target values for *E. faecalis* and *E. faecium* infections

The PK/PD target values associated with treatment outcomes and AKI were analyzed separately for *E. faecalis* and *E. faecium* (Table S2). For *E. faecium*, ROC curve analysis identified significant cut-off values for both *C*_min_ and AUC_24h_ in predicting clinical efficacy and AKI (Fig. 3C and D). Based on clinical remission, the optimal thresholds were determined to be *C*_min_ ≥ 9.97 mg/L (sensitivity: 76.4%; specificity: 64.0%) and AUC_24h_ ≥ 424.40 mg·h/L (sensitivity: 65.3%; specificity: 72.0%). For AKI prediction, the cut-off values were *C*_min_ ≥ 18.01 mg/L (sensitivity: 59.1%; specificity: 84.0%) and AUC_24h_ ≥ 562.80 mg·h/L (sensitivity: 68.2%; specificity: 84.0%). To facilitate clinical application, these target values were rounded to integers, resulting in a therapeutic window of AUC_24h_ 425–560 mg·h/L and *C*_min_ 10–18 mg/L. In contrast, for *E. faecalis*, no significant PK/PD cut-off values were identified for predicting efficacy (*P > 0.05*); however, a weak association between *C*_min_ and AKI was observed (*P = 0.039*). The ROC curves for *E. faecalis* were not clinically actionable, likely due to the limited sample size or uneven distribution of isolates, which precluded the derivation of meaningful thresholds.

## DISCUSSION

This study systematically analyzed 154 patients with enterococcal infections. Multivariate analysis identified an AUC_24h_ > 380 mg·h/L as an independent predictor of clinical efficacy, while an AUC_24h_ > 540 mg·h/L was identified as an independent risk factor for AKI. Additionally, the SOFA score, age over 60 years, and the presence of intra-abdominal infection were confirmed as significant determinants of clinical outcomes. Building on these findings, we further determined the specific vancomycin exposure thresholds through ROC curve analysis. For enterococcal infections, we propose a therapeutic target range of AUC_24h_ 370–540 mg·h/L and *C*_min_ 10–14 mg/L. For *E. faecium* infections, the target range is AUC_24h_ 425–560 mg·h/L and *C*_min_ 10–18 mg/L. These ranges optimally balance therapeutic efficacy with the risk of nephrotoxicity, providing critical evidence to support individualized precision dosing for enterococcal infections.

The overall efficacy rate of vancomycin in treating *Enterococcus* infections in this study was 72.1%, which was lower than the reported efficacy rate of 82.7% previously (27). It is notable that the therapeutic effect of non-invasive bloodstream infections was significantly better than that of invasive bloodstream infections. Invasive bloodstream infections require vigilance for treatment failure. In the study by Piyawadee Tangvichitrerk et al., there was no significant difference in clinical efficacy and bacterial clearance between the AUC > 420 group and the AUC < 420 group in patients with *Enterococcus* bloodstream infections (28). This suggests that merely increasing the vancomycin concentration or AUC is not sufficient to improve the clinical efficacy of patients with bloodstream infections. This result is related to the fact that *Enterococcus* is prone to form biofilms in bloodstream infections, which restricts drug penetration (29, 30), and may also be related to the host’s immune status (31). The multivariate analysis in this study showed that AUC_24h_ > 380.00 mg·h/L was an independent predictor of clinical efficacy, which is comparable to the treatment goal for MRSA infections (AUC/MIC ≥ 400.00) (32). This suggests that the PK target threshold for different pathogens may vary, and the target AUC_24h_ for *Enterococcus* infections with vancomycin is lower than that for MRSA infections, and the exposure amount required to reach each target is crucial for ensuring therapeutic efficacy. The observed difference in the AUC_24h_ threshold between enterococcal and MRSA infections aligns with the structural differences discussed in the Introduction. Specifically, the distinct peptidoglycan architecture of *Enterococcus*, characterized by a higher content of low-branched cross-bridge peptides, may impede the penetration efficiency of vancomycin. This suggests that a different exposure target may be necessary to achieve comparable bacterial killing at the infection site. This finding provides important clinical value for guiding the effective treatment of *Enterococcus* bloodstream infections with vancomycin. This study believes that the vancomycin treatment regimen for *Enterococcus* bloodstream infections needs to be optimized, and it is recommended to use vancomycin in combination or extend the treatment course.

The 30-day all-cause mortality rate of the patients in this study was 14.3%, significantly lower than the mortality rate range of 13.4%–26.7% reported in the literature (27, 31, 33–37). This difference may be related to multiple factors such as patient population characteristics, severity of the disease, and treatment management strategies. From the perspective of the patient population, a heavy burden of underlying diseases and a high degree of organ failure are the key factors contributing to the increased mortality rate (31, 36). From the perspective of treatment management, the timely guidance of medication adjustments by the clinical pharmacists in this study can ensure the achievement of target trough concentrations or AUC_24h_ through TDM, which may be one of the reasons for the lower mortality rate. It is worth noting that even reaching a high AUC_24h_ (580.00 mg·h/L) still cannot improve the prognosis of critically ill elderly patients, which is consistent with previous research results (31). This phenomenon suggests that disease severity indicators such as SOFA score have an independent impact on prognosis, independent of vancomycin exposure parameters; for critically ill patients, simply optimizing drug exposure levels is not sufficient to improve the outcome; a comprehensive treatment strategy including infection source control and organ function support is needed. The results of this study provide important insights for clinical practice. When using vancomycin to treat *Enterococcus* infections, individualized administration should be implemented under the guidance of TDM, and comprehensive management of underlying diseases and organ functions should be emphasized, especially for the critically ill patient population.

The 14-day bacterial clearance rate of vancomycin was 77.9%, which was much lower than the 92.3% reported in the literature (27). The clearance difficulty of *Enterococcus* infections is closely related to the infection site. For complex infections, the clearance rate is often lower due to issues such as biofilm formation and limited drug penetration (29, 30). This study found that intra-abdominal infections adversely affect bacterial clearance. The incidence of abdominal infections was 39.0% (60/154), with a notably high prevalence, and the proportion of complex infections was relatively high, which may contribute to a decrease in the bacterial clearance rate. In addition, immunosuppression (such as concurrent malignant tumors) would reduce the bacterial clearance ability (7, 31). The proportion of concurrent malignant tumors in this study was 39.6% (61/154), which might also be one of the reasons for the low bacterial clearance rate. This study further confirmed the significant correlation between vancomycin *C*_min_ and bacterial clearance rate. When *C*_min_ is greater than 11.00 mg/L, it is beneficial to increase the bacterial clearance rate, which is higher than the minimum effective concentration in this study (*C*_min_ ≥ 9.97 mg/L). As a time-dependent antibacterial drug, the complete clearance of bacteria requires maintaining a drug concentration higher than the MIC of the pathogenic bacteria for a longer period of time, enhancing the drug penetration ability and inhibiting bacterial regrowth, thereby significantly improving the thoroughness of bacterial clearance. This result further confirms the importance of optimizing the monitoring of vancomycin trough concentration based on PK characteristics for improving clinical efficacy.

The incidence of AKI in this study was 20.8%, which was lower than the reported incidence of 21.5% (36) and 50% (28). The occurrence of AKI was significantly correlated with the SOFA score and AUC_24h_ > 540.00 mg·h/L. Although the proportion of patients using vancomycin in combination with nephrotoxic antibacterial drugs was relatively high in this study, through individualized adjustment of the antibacterial treatment plan through TDM, the incidence of AKI did not significantly increase. However, this might also be the reason that the vancomycin nephrotoxic threshold in this study was lower than that reported in other studies, such as Rybak et al. (38) for MRSA infections with AUC_24h_ > 700 mg·h/L, and Piyawadee et al. for patients with *Enterococcus* bloodstream infections using vancomycin combined with nephrotoxic drugs, with an AUC_24h_ > 650 mg·h/L (28). It is important to clarify that nephrotoxicity is a drug-specific effect and, theoretically, should not vary by the type of infecting pathogen. Consequently, the lower threshold observed in our cohort is unlikely to be attributable to the pathogen itself. Instead, it likely reflects differences in the characteristics of the patient population—particularly the higher disease severity, indicated by a median SOFA score of 3, and the frequent use of concomitant nephrotoxic antibiotics in our study. These factors may have increased patients’ susceptibility to vancomycin-induced kidney injury, thereby effectively lowering the exposure threshold at which AKI occurs. This interpretation is further supported by the multivariate analysis, which identified the SOFA score as an independent risk factor for AKI. This further emphasizes the importance of implementing drug concentration monitoring and dynamic assessment of renal function for critically ill patients. This study provides evidence-based support for the individualized use of vancomycin in patients with severe *Enterococcus* infections and has important guiding value for the prevention and control of AKI in clinical practice.

This study determined the PK/PD target values for vancomycin treatment of *Enterococcus* infections through ROC curve analysis and established a dual-threshold therapeutic window based on AUC_24h_: the efficacy threshold ≥368.30 mg·h/L (to ensure antibacterial effect) and the safety threshold <535.00 mg·h/L (to prevent nephrotoxicity), thereby defining the optimized therapeutic range. This discovery broke through the limitations of existing guidelines that mainly relied on MRSA infection data (11, 19) and, for the first time, provided evidence-based support for precise vancomycin administration for *Enterococcus* infections. The target range of *C*_min_ (10–14 mg/L) determined by the study was highly consistent with the 10–20 mg/L recommended by the International Society for Infectious Diseases (11), further supporting the reliability of trough concentration monitoring in clinical practice. Based on the research data, we proposed the optimized vancomycin treatment target values for patients with *Enterococcus* infections: AUC_24h_ 370–540 mg·h/L combined with *C*_min_ 10–14 mg/L. This strategy is particularly suitable for elderly patients with unstable renal function and can effectively balance efficacy and risk of nephrotoxicity. In clinical application, we suggest routine TDM for patients with *Enterococcus* infections and individualized administration. For critically ill patients with a SOFA score ≥2, the drug concentration and renal function monitoring frequency should be increased to optimize treatment and reduce the risk of AKI. This study provides evidence-based support for precise vancomycin administration for *Enterococcus* infections and has important guiding value for optimizing clinical practice. In interpreting ROC findings, it is important to acknowledge that the predictive performance of vancomycin exposure parameters, as indicated by the moderate AUC_24h_ values in our ROC analyses (ranging from 0.67 to 0.722 for efficacy and AKI prediction), highlights the multifactorial nature of clinical outcomes in enterococcal infections. Although drug exposure is a critical modifiable factor, it functions within a complex clinical context in which patient-specific factors—such as disease severity, infection site, and comorbidities—exert substantial influence. Consequently, rather than acting as standalone predictors, the identified PK/PD thresholds should be interpreted as targets for optimizing drug exposure within a comprehensive treatment strategy that encompasses source control and organ support. This perspective is consistent with the principles of therapeutic drug monitoring, where achieving target concentrations is necessary but not sufficient for favorable outcomes.

However, species-specific analysis revealed distinct thresholds for *E. faecium*—the predominant pathogen in our cohort—with a narrower therapeutic window of AUC_24h_ 425–560 mg·h/L and *C*_min_ 10–18 mg/L. The higher upper limits for *C*_min_ (18 mg/L vs 14 mg/L) and AUC_24h_ (560 mg·h/L vs 540 mg·h/L) in the *E. faecium*-specific analysis suggest that this pathogen may tolerate slightly higher drug exposures before AKI onset, allowing for safely increased trough concentrations under close renal monitoring. Based on these findings, we propose a stratified approach to vancomycin TDM for enterococcal infections. When species identification is pending or empirical therapy is initiated, the broader window derived from all *enterococci* (AUC_24h_ 370–540 mg·h/L; *C*_min_ 10–14 mg/L) serves as a safe and effective initial target, balancing efficacy (AUC_24h_ ≥ 370 mg·h/L) with minimized AKI risk (AUC_24h_ ≤ 540 mg·h/L). Once *E. faecium* is confirmed as the causative pathogen, transition to the species-specific window (AUC_24h_ 425–560 mg·h/L; *C*_min_ 10–18 mg/L) is recommended. For *E. faecalis* infections, where no efficacy thresholds were identified, adherence to the more conservative combined cohort targets or standard susceptibility breakpoints may suffice. This stratified approach tailors vancomycin exposure to both pathogen and clinical context, optimizing therapeutic individualization.

The innovation of this study lies in the first establishment of specific vancomycin PK/PD target values for *Enterococcus* infections, and the results were verified through multiple models (such as ROC analysis and sensitivity analysis) to ensure their reliability. However, there are also some limitations, including the possible introduction of selection bias due to the single-center retrospective study design. The relatively limited sample size may affect the efficacy of subgroup analyses. Although clinical efficacy assessment has been refined, it remains partly subjective. Furthermore, the lack of systematic recording and analysis of concomitant nephrotoxic drug use (e.g., analgesics and antihypertensive drugs) presents another limitation. These factors may confound the relationship between AKI and vancomycin exposure. Furthermore, the MIC of vancomycin for specific enterococcal isolates is a critical determinant of therapeutic success, as it directly influences the attainment of optimal AUC_24h_/MIC ratios. Variability in MIC distribution among isolates—even within the susceptible range—may impact both efficacy and toxicity outcomes. The narrow MIC range in our study (all isolates MIC ≤ 2 mg/L) may have limited our ability to detect the independent predictive value of the AUC_24h_/MIC ratio. Therefore, future multicenter studies with larger sample sizes should incorporate MIC data to perform subgroup analyses stratified by AUC_24h_/MIC ratios, along with more complex modeling techniques (such as CART or machine learning methods), thereby enabling more precise target identification and personalized vancomycin dosing strategies for enterococcal infections, as well as exploring potential interactions among variables.

### Conclusion

This research formulates an optimized dosing strategy for vancomycin in the treatment of *Enterococcus* infections. It confirms that individualized dosing guided by TDM is of paramount importance.

The study proposes an ideal therapeutic target window, with an AUC_24h_ ranging from 370 to 540 mg·h/L and a *C*_min_ from 10 to 14 mg/L. For *E. faecium*, the therapeutic target range was defined as AUC_24h_ 425–560 mg·h/L and *C*_min_ 10–18 mg/L. Based on species-specific differences, a stratified management approach is recommended. When species identification is pending, the broader target range (AUC_24h_ 370–540 mg·h/L; *C*_min_ 10–14 mg/L) serves as an initial target to balance efficacy and safety. Once *E. faecium* is confirmed, the species-specific window (AUC_24h_ 425–560 mg·h/L; *C*_min_ 10–18 mg/L) may be adopted under close renal monitoring. For *E. faecalis*, where no efficacy threshold has been identified, adherence to the conservative combined cohort targets or standard susceptibility breakpoints is advised.

This target range not only ensures antibacterial effectiveness but also mitigates the risk of nephrotoxicity, thus providing a crucial basis for dosage adjustment in clinical practice. These research findings address the deficiencies in current guidelines for the treatment of *Enterococcus* infections and hold substantial clinical significance for enhancing patient prognosis. However, it is necessary to further validate the general applicability of this target range through multicenter prospective studies. Additionally, there is a need to explore optimal dosing regimens for diverse patient subgroups, such as those with renal insufficiency and elderly patients.

## SUPPLEMENTAL MATERIAL

Supplemental material spectrum.00937-26-s0001.docx Fig. S1 and S2; Tables S1 and S2.

ASM does not own the copyrights to Supplemental Material that may be linked to, or accessed through, an article. The authors have granted ASM a non-exclusive, world-wide license to publish the Supplemental Material files. Please contact the corresponding author directly for reuse.
