# Supplementary material for: PK/PD study of vancomycin in enterococcal infections: efficacy and safety assessment under the guidance of TDM
Source: Microbiol Spectr. 2026 Jun 15;14(7):e00937-26. doi: 10.1128/spectrum.00937-26 (PMC13339812; doi:10.1128/spectrum.00937-26)
Supplement: Supplemental material — Fig. S1 and S2; Tables S1 and S2. [file spectrum.00937-26-s0001.docx]

**
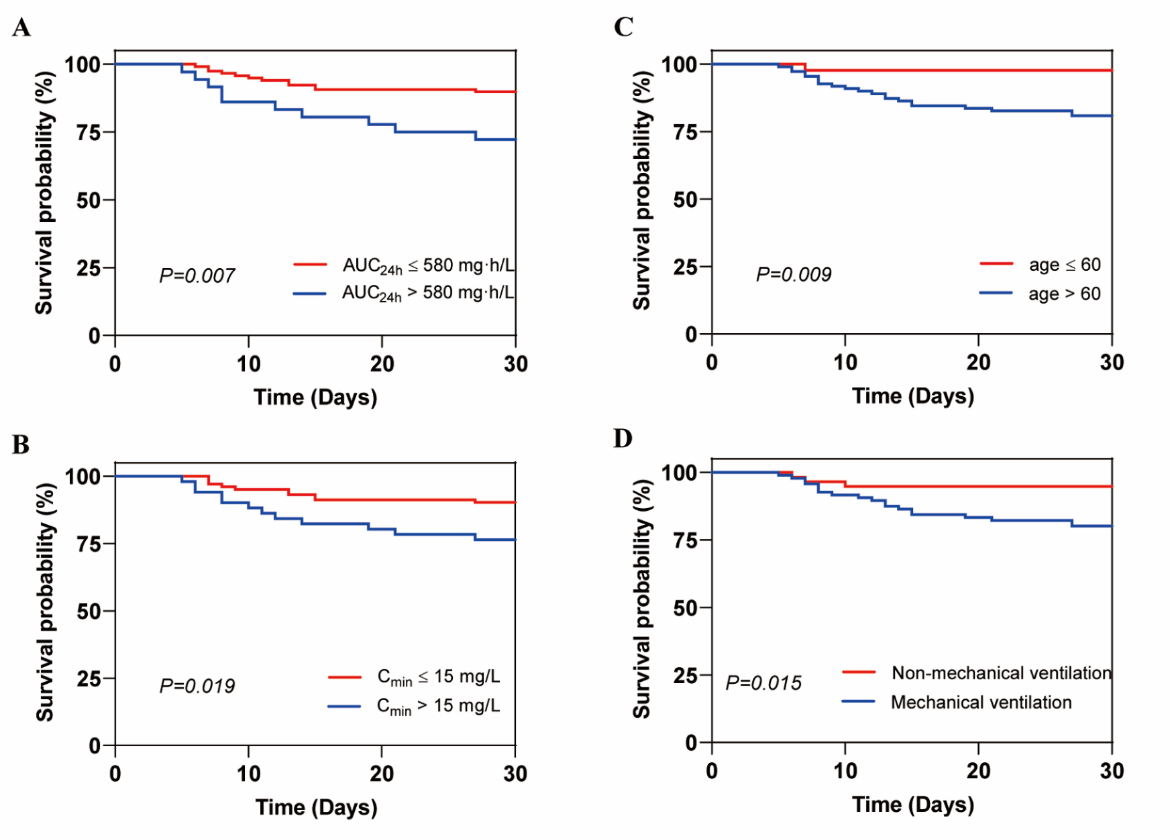
**

**Figure S1** Kaplan-Meier survival estimates for 30-day all-cause mortality.

(A) AUC_24h_ ≤ 580 and AUC_24h_ > 580 mg·h/L (*P* < 0.01). (B) C_min_ ≤ 15 and C_min_ > 15 mg/L (*P* < 0.01). (C) age ≤ 60 and age > 60 (*P* < 0.01). (D) Non-mechanical ventilation and Mechanical ventilation (*P* < 0.05).

C_min_, Trough concentration; AUC_24h,_ 24-hour area under the concentration-time curve at steady state.


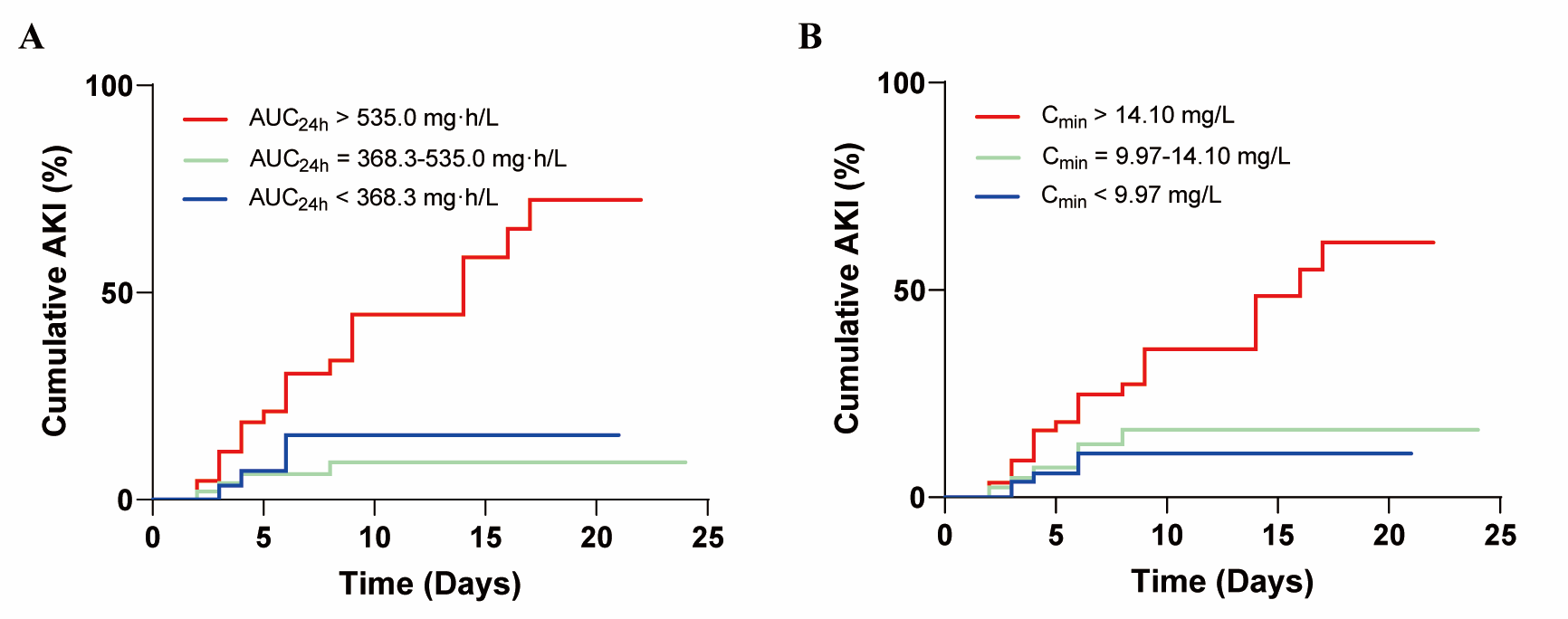


**Figure S2** Kaplan-Meier estimates of the incidence of AKI after vancomycin administration.

(A) AUC_24h_< 368.30，368.30 - 535.00 and > 535.00 mg·h/L. (B) C_min_< 9.97, 9.97 - 14.10 and > 14.10 mg/L.

C_min_, Trough concentration; AUC_24h_; 24-hour area under the concentration-time curve at steady state.

**Table S1** Analysis of factors affecting 14-Day-bacterial clearance during vancomycin treatment

| **Parameters** | **Bacterial clearance group（n=120）** | **Non-bacterial clearance group (n=34)** | **OR (95%CI)** | ***P* value** |
| --- | --- | --- | --- | --- |
| **Univariate analysis** |  |  |  |  |
| Age, years, median (IQR) | 71.0 (59.25, 79.75) | 66.5 (59.25, 77.25) | 1.007 (0.985 - 1.03) | 0.539 |
| **Comorbidities, n (%)** |  |  |  |  |
| Heart disease | 35 (29.2) | 10 (29.4) | 0.988 (0.428 - 2.28) | 0.978 |
| Diabetes | 32 (26.7) | 6 (17.6) | 1.303 (0.802 - 2.116) | 0.285 |
| Hypertension | 50 (41.7) | 16 (47.1) | 0.93 (0.72 - 1.2) | 0.575 |
| Kidney diseases | 17 (14.2) | 1 (2.9) | 1.528 (0.914 - 2.553) | 0.106 |
| Liver diseases | 18 (15.0) | 3 (8.8) | 1.128 (0.872 - 1.459) | 0.360 |
| Malignancy | 45 (37.5) | 16 (47.1) | 0.937 (0.824 - 1.065) | 0.316 |
| **Test index before Vancomycin treatment** |  |  |  |  |
| WBC, 10^9^/L, median (IQR) | 12.38 (8.65,18.57) | 13.16 (8.72,15.59) | 1.031 (0.979 - 1.084) | 0.248 |
| CRP, mg /L, median (IQR) | 105 (43,160) | 101 (56,160) | 0.999 (0.992 -1.005) | 0.685 |
| PCT, ng /mL, median (IQR) | 1.62 (0.24,7.51) | 1.16 (0.26,6.51) | 1.016 (0.986  **-** 1.047) | 0.307 |
| SOFA, median (IQR) | 3 (1,5) | 3 (1,5) | 1.092 (0.942  **-** 1.266) | 0.242 |
| Mechanical ventilation, n (%) | 79 (65.8) | 17 (50.0) | 1.927 (0.891  **-** 4.165) | 0.095 |
| CRRT, n (%) | 16 (13.3) | 3 (8.8) | 1.590 (0.435  **-** 5.814) | 0.483 |
| Surgery, n (%) | 95 (79.2) | 26 (76.5) | 1.169 (0.472 **-** 2.895) | 0.735 |
| ICU, n (%) | 34 (28.3) | 13 (38.2) | 0.639 (0.288 **-** 1.418) | 0.270 |
| **Infection type, n (%)** |  |  |  |  |
| Bloodstream infection | 47 (39.2) | 10 (29.4) | 1.545 (0.678 **-** 3.522) | 0.300 |
| Pulmonary infection | 7 (5.8) | 4 (11.8) | 0.682 (0.357 **-** 1.301) | 0.245 |
| Wound infection | 9 (7.5) | 3 (8.8) | 0.943 (0.598 **-** 1.486) | 0.800 |
| Urinary tract infection | 37 (30.8) | 7 (20.6) | 1.114 (0.928 **-** 1.339) | 0.247 |
| Intra-abdominal infection | 42 (35.0) | 18 (52.9) | 0.884 (0.778 **-** 1.006) | 0.061 |
| **Vancomycin Therapeutic regimen** |  |  |  |  |
| Single dose of vancomycin, mg/kg, median (IQR) | 8.33 (7.61,10.00) | 8.70(8.33,10.27) | 0.930 (0.82 **-** 1.056) | 0.263 |
| Daily dose, mg /kg, median (IQR) | 25.00 (17.10,30.00) | 24.80 (18.06,30.00) | 0.9995 (0.96 **-** 1.04) | 0.982 |
| **Concomitant antibiotics, n (%)** |  |  |  |  |
| Imipenem cilastatin | 18 (15.0) | 5 (14.7) | 1.024 (0.35 **-** 2.994) | 0.966 |
| Meropenem | 43 (35.8) | 11 (32.4) | 1.168 (0.52 **-** 2.624) | 0.707 |
| Piperacillin tazobactam | 22 (18.3) | 4 (11.8) | 1.684 (0.538 **-** 5.271) | 0.371 |
| Levofloxacin | 14 (11.7) | 0 (0) | 518171176.009 (0 **-** 0) | 0.999 |
| Amikacin | 3 (2.5) | 1 (2.9) | 0.846 (0.085 **-** 8.405) | 0.887 |
| Fosfomycin | 3 (2.5) | 2 (5.9) | 0.414 (0.066 **-** 2.583) | 0.345 |
| Polymyxin | 4 (3.3) | 4 (11.8) | 0.259 (0.061 **-** 1.095) | 0.066 |
| MIC, median (IQR) | 0.75 (0.5,1.0) | 0.75 (0.5,1.0) | 0.965 (0. 458 **-** 2.032) | 0.924 |
| **TDM, median (IQR)** |  |  |  |  |
| C_min_, mg/L | 12.2 (8.9,18.4) | 9.7 (6.1,15.0) | 1.091 (1.019 **-** 1.169) | **0.013** |
| C_min_> 11 vs. ≤ 11, mg/L, n (%) | 74 (61.7) | 13(38.2) | 2.599 (1.187 **-** 5.689) | **0.017** |
| AUC_24h_, mg·h/L | 443.4 (336.2,581.9) | 336.2 (254.1,539.9) | 1.003 (1.000 **-** 1.005) | **0.029** |
| AUC_24h_ > 405 vs. ≤ 405, n (%) | 71 (59.2) | 13 (38.2) | 2.341 (1.071 **-** 5.114) | **0.033** |
| AUC /MIC | 636.3 (388.86,939.07) | 505.82 (290.44,664.16) | 1.001 (1.000 **-** 1.002) | 0.102 |
| C_max_, mg/L | 23.64 (18.32,31.49) | 19.475 (14.77,30.05) | 1.039 (0.994 **-** 1.085) | 0.088 |
| **Pathogenic bacteria species, n (%)** |  |  |  |  |
| *E. feacalis* | 46 (38.3) | 18 (52.9) | 0.553 (0.257 **-** 1.19) | 0.130 |
| *E. faecium* | 78 (65.0) | 19 (55.9) | 1.211 (0.822 **-** 1.783) | 0.333 |
| *E. avium* | 6 (5.0) | 1 (2.9) | 1.202 (0.587 **-** 2.463) | 0.615 |
| *E. gallinarum* | 2 (1.7) | 2 (5.9) | 0.722 (0.438 **-** 1.189) | 0.201 |
| **Multivariate analysis** |  |  |  |  |
| Intra-abdominal infection |  |  | 0.444 (0.199 **-** 0.991) | **0.048** |
| Mechanical ventilation |  |  | 2.040 (0.913 **-** 4.560) | 0.082 |
| C_min_> 11 vs. ≤ 11 |  |  | 2.566 (1.151 **-** 5.718) | **0.021** |

For all binary comparisons presented as "≥/＞X vs. ≤/＜Y" in the tables, only the number and percentage of isolates in the higher category (≥/＞X) are shown. The remaining isolates fall into the complementary lower category (≤/＜Y), with percentages calculated based on the total number of strains in each respective group.

IQR, interquartile range; eGFR, estimated glomerular filtration rate; SOFA, sequential organ failure assessment; CRRT, continuous renal replacement therapy; TDM, Therapeutic drug concentration monitoring; C_min_, trough concentration; AUC_24h_, 24-hour area under the concentration-time curve at steady state; C_max_, peak concentration; MIC, minimal inhibitory concentration; peak concentration.

**Table S2** Analysis of PK/PD target values for *E. faecalis* and *E. faecium* infections

| **Pathogenic bacteria species** | **Effective （n）** | **Failure （n）** | **C_min_ cut-off value** | ***P* value** | **AUC_24h_ cut-off value** | ***P* value** |
| --- | --- | --- | --- | --- | --- | --- |
| *E. feacalis* | 42 | 22 | - | 0.292 | - | 0.562 |
| *E. faecium* | 72 | 25 | 9.97 | 0.001 | 424.4 | 0.004 |
|  | **AKI （n）** | **Non-AKI （n）** | **C_min_ cut-off value** | ***P* value** | **AUC_24h_ cut-off value** | ***P* value** |
| *E. feacalis* | 10 | 54 | - | 0.039 | - | 0.104 |
| *E. faecium* | 22 | 75 | 18.01 | 0.002 | 562.8 | 0.002 |

C_min_, trough concentration; AUC_24h_, 24-hour area under the concentration-time curve at steady state;
